# The Institutional Library

**Published:** 1917-08-04

**Authors:** 


### THE INSTITUTIONAL LIBRARY.

### INTEMPERATE TEMPERANCE.

IT has often been remarked that the writer who devotes his pen to the spread of temperance and to a combat against drunkenness is strangely liable to drift into invective. In the process of accumulating facts and sifting evidence a kind of frenzy often seems to descend upon him. Facts acknowledged by every civilised being lie ready to his hand. But yet, sinking sometimes to a level of fanaticism, he will choose to embroider his thesis with inaccurate statements, to pick quarrels with unoffending persons, and to rouse against himself and his cause an astounding personal hostility. A little pamphlet* by Dr. Mercier, which has been recently put forth by a body which calls itself the "True Temperance Association," bears queer testimony to the acrimony which the advocates of total abstinence are capable of rousing. It has to be admitted that the "moderate drinker" himself occasionally exhibits a very touchy disposition, and Dr. Mercier's well-known raciness of style makes his indignation a very amusing affair. Dr. Mercier, apart from the merits of the controversy, makes the very effective point, and the most original comment in the pamphlet, that the total abstinence party, while vigorously condemning alcohol, has never made any attempt to get to the reason why people drink it. Is the habit due to madness or to badness? Obviously this must be decided before we are in a position to answer the question—Should the curate or the constable be called in? He scores a good point when he remarks that "if we are to abjure alcohol because it is injurious to cabbages, it would be a good plan to drink diluted sewage which is so beneficial to them," and few would deny his contention that "the absurd exaggeration of the teetotal fanatic brings discredit on their cause and makes the people indifferent to their intemperate outcries. In all this we have no quarrel with the protesting "moderate." But none the less a quarrel we have with him. Fanaticism, he quite plainly reveals, does not lie exclusively with the total abstainer, and in the quest of truth fanaticism invariably leads to a quagmire. What are we to say of a writer who exclaims "How can any teetotaller have the hardihood to maintain that drunkenness has increased, is increasing, and is as bad as ever? The fact is undeniable and indisputable that the English . . . are now a sober nation." That this is an altogether too sanguine view of the situation even the True Temperance Association must have come to recognise since the exigencies of the military demands for labour have led to a survey of English sobriety. But even without the illustration lately afforded of the drinking habits prevalent among large sections of the population, any table of statistics on the subject reveals that though drinking to excess has gone out of fashion in certain classes in public, it still sweeps away nearly as many lives as formerly. The death-rate per million from alcoholism was 55 in 1889, it was 55 in 1908, with strange fluctuations in between. The average number of persons per 1,000 proceeded against for drunkenness was 4.28 between 1857-1861, and had risen to 6.51 by 1902-1906. On these figures the writer in the "Encyclopædia Britannica" observes: "That the increase was partly due to more efficient police control is probable, but that this is not a complete explanation of the figures is made evident by an analysis of the general statistics of crime during the same period, from which it may be seen that while crime generally (excluding drunkenness) decreased 28 per cent. in England and Wales since 1857-1861, drunkenness increased 51 per cent." It would certainly become the advocates of "True Temperance" to look at such figures before denouncing the teetotaller when he presses for renewed efforts against drunkenness. And what can be said to the further contention of the temperate writer? "There has never been a distinguished originator in any branch of art who did not take alcohol. . . . There is no great artist in any branch of creative art—poet, painter, novelist, dramatist, musician, or what not—who has not enjoyed his glass of wine or of whiskey if he could get it." Surely this Samson has delivered himself bound into the hands of his enemies. Had he peered through the grating of prejudice into the Elysian Fields the first person he might have beheld walking beside the still waters reserved for the total abstainer was—Shelley. "No great artist in any branch of creative art . . ."! "Good heavens!" the total abstainer may with reason inquire, "where are these moderate drinkers brought up?"

* *The Intemperance of Total Abstinence.* By C. A. Mercier, M.D., F.R.C.P., etc. (The True Temperance Association. Price 2d.)

### THE MAGDALEN HOSPITAL.

**The Magdalen Hospital.** By the Rev. H. F. B. COMPSTON, M.A. (London: Society for Promoting Christian Knowledge. 1917. Pp. 237. Price 7s. 6d. net.)

MR. COMPSTON'S book, which bears the second title, "A Story of a Great Charity," is very human, and is in keeping with the humane principles which are rightly claimed as actuating the administration from the foundation. The plan of the founder, Mr. Robert Dingley, evidently had this quality in view, for at the first meeting of the committee it was resolved that "the plan now under consideration shall be maturely examined and rendered more constitutional and prudent, humane and practicable."

"Noble and extensive are the Charities already established in this City: Unfortunate Females seem the only Objects that have not catched the attention of public Benevolence." This was the theme of a pamphlet embracing Mr. Dingley's idea; he quickly won the support of other City men, and with most businesslike expedition the charity was formed, housed, and caring for its first "objects" within a few months during the year 1758. Such was the birth of the first institution of its kind. Over four hundred charities more or less similar to the Magdalen bear witness to the example set in 1758, the prototype of these having since that year, successfully in most cases, dealt with 14,000 penitents.

With a wealth of detail and richness of collateral illustration in the shape of documents, of portraits, of topographical engravings, Mr. Compston pilots us through a century and a half of institutional history, taking care that the history is accompanied throughout by a sufficiency of description of contemporary manners and events to make the whole doubly interesting. To institutional readers the history of Magdalen is well worthy of study, but in the form in which it is presented it can easily attract and hold the attention of a much wider circle of readers. As one illustration of how Mr. Compston can be analytical without boring his readers, one might refer to his examination of the difference of opinion as to whether Saint Mary Magdalen was or was not a fallen woman; the evidence is succinctly marshalled, and the conclusion seems to be that, although there is little or no testimony to support the case that might have been prepared by the devil's advocate, the saint cannot complain of the personal effect of any misapprehension, as the damages are considerably outweighed by the enormous amount of good effected through the legend of the fallen woman who became a saint.

The Magdalen had for its first home houses in Prescott and Chambers Streets, Whitechapel, which had just been vacated by the London Hospital prior to its installation on the present site. This neighbourhood was known as Goodman's Fields, and here was situated the Magdalen Hospital until its first flitting, in 1772, to "near the New Bridge from Black Fryers on the Southwark Side." The foundation-stone of the new building was laid in 1769, but owing to building difficulties the hospital was not opened until three years later. There is, however, a coincidence about the date, as it was 100 years later (in 1869) that the institution moved to its present home in Streatham.

On one night in 1762 forty women were arrested and taken to Bridewell; eleven were whipped, some were sent to their friends, and one to the Magdalen Hospital. This throws a very faint light on how the hospital obtained its patients or penitents; there were no social workers in those days who could direct errant steps towards the Magdalen. But some, even then, came of their own will. Ann Blore, a native of Ashburn, Derbyshire, was the first; two more were promised admission as soon as they "were cured of disease." "Mary Truman was rejected, being no Prostitute." These were some of the first applicants who, in the words of our chronicler, "had taken the wrong turning; some of them, no doubt more sinned against than sinning, turned their faces to the light. They were the pioneers of an army of more than fourteen thousand penitents who have found a home and friends and helpers in the Magdalen. All honour to Robert Dingley and his committee for the good work they set going in 1758."

The book includes complete lists of the chief officers from the foundation of the Charity, and essential financial and other statistics. There is a foreword by the Archbishop of Canterbury.

**An Index of Symptoms, with Diagnostic Methods.** By RALPH WINNINGTON LEFTWICH, M.D. Sixth Edition. (London: Smith, Elder and Co. 1917. Pp. 555 + xii. Price 10s. 6d. net.)

WE have writen in appreciative terms of the earlier editions of this book and the latest issue merits an equal commendation. In truth, the best of testimonies to its value is its success, and this more particularly seeing that its aim is a strictly practical one. Medicine has no such simple formula as "one symptom, one disease." On the contrary, each symptom has many possible interpretations, and the aim of Dr. Leftwich's Index is to keep these possibilities before the eye and judgment of the practitioner. The work is carried out with admirable thoroughness and diligence, and each new edition has borne evidence of the watchful zeal of the author. Thus in the present issue have been added a section dealing with the main features of some seventy more or less rare diseases and a list of Eponymous Signs as these are associated with clinical medicine. We have never been happy about Dr. Leftwich's illustrations of sphygmograms, and their re-appearance does not reconcile us to them. But we are indebted to him in many directions, and he will forgive us when we halt at his pulse tracings.

**National Health Insurance Commission (England).** Reports of Inquiries and Appeals under the National Health Insurance (Medical Benefit) Regulations (England), 1913.

A BLUE-BOOK under the above title, and with the further description Volume I., has recently been issued. It contains reports of *all* inquiries upon medical practitioners, and upon persons, firms, and corporate bodies under contract to supply drugs and appliances, which have been held since medical benefit commenced on January 1, 1913, up to the end of the year 1916. The number of inquiries upon medical practitioners is only twenty-one, and the number upon those supplying drugs and appliances was only seven. This is a truly remarkable record for four years' work, considering the wide ramifications of medical benefit under the National Insurance Acts and the large number of doctors and chemists who have undertaken panel service. The reports do not disclose the names of the practitioners nor of the chemists, but they give the salient facts and the decisions arrived at. They do not form very pleasant reading, because, in the vast majority of cases, the charges made were substantiated and the names of the doctors and chemists have mostly been directed to be removed from the panel. The object of the publication was, doubtless, to preserve in a permanent form a record of the work done, but there is very little in the Reports that would be of assistance in the way of precedent in deciding future cases. As a deterrent to malpractice or breach of the medical-benefit regulations the publication is quite ineffective. It is somewhat astonishing that the present moment, when the supply of paper is so short, should have been chosen to issue the volume.

**An Outline of the Medical Treatment of Venereal Diseases in Women.** By MARY SCHARLIEB, M.D., M.S., and MORNA L. RAWLINS, M.B., B.S. (London: The National Council for Combating Venereal Disease. 1917. Pp. 14. Price 2d.)

THIS pamphlet of fourteen pages makes a brave show. It boasts a sonorous title and claims the patronage of a dignified philanthropic organisation. To its preparation two practitioners have devoted themselves, and—somehow it seems an anti-climax—the price is twopence! What purpose is to be served by the pamphlet we cannot imagine. It is too technical for the general public, and its slender proportions will hardly attract the medical practitioner. The information it affords is necessarily limited and incomplete, and as a literary exercise its merits are microscopic. The National Council might surely have spent money and influence to better purpose. Anyhow, so far as we are concerned, we advise our readers to spare their coppers.

**General Service Hints for V.A.D. Members.** By Mrs. THORNTON COOK, revised by Miss Lilian Deunler. With Preface by Mrs. Katharine Furse, Commander-in-Chief, Women's V.A.D.s. (London: The Scientific Press, Ltd. Price 1s. 3d. net.)

EXTRA pains have been taken to render this little manual accurate and helpful. We have seen no publication more suited for putting into the hands of those (still rather a large class, it would seem) who want to be employed in one capacity or another in a military hospital. The chapter on "Daily Routine" and "Army Forms" gives concise information nowhere else accessible to the beginner, so far as we are aware, and will prove a sure guide to a very tangled labyrinth.

